# Pylephlebitis: A Systematic Review on Etiology, Diagnosis, and Treatment of Infective Portal Vein Thrombosis

**DOI:** 10.3390/diagnostics13030429

**Published:** 2023-01-25

**Authors:** Lisa Fusaro, Stefano Di Bella, Paola Martingano, Lory Saveria Crocè, Mauro Giuffrè

**Affiliations:** 1 Department of Medical, Surgical and Health Sciences, University of Trieste, 34149 Trieste, Italy; 2 Infectious Disease Department, Azienda Sanitaria Universitaria Giuliano-Isontina (ASUGI), 34128 Trieste, Italy; 3 Departmet of Radiology, Azienda Sanitaria Universitaria Giuliano-Isontina (ASUGI), 34128 Trieste, Italy; 4 Liver Clinic, Azienda Sanitaria Universitaria Giuliano-Isontina (ASUGI), 34128 Trieste, Italy

**Keywords:** pylephlebitis, portal vein, portal vein thrombosis, *Escherichia coli*, *Bacteroides*, *Streptococcus*, anticoagulant, diverticulitis, appendicitis

## Abstract

Pylephlebitis, defined as infective thrombophlebitis of the portal vein, is a rare condition with an incidence of 0.37–2.7 cases per 100,000 person-years, which can virtually complicate any intra-abdominal or pelvic infections that develop within areas drained by the portal venous circulation. The current systematic review aimed to investigate the etiology behind pylephlebitis in terms of pathogens involved and causative infective processes, and to report the most common symptoms at clinical presentation. We included 220 individuals derived from published cases between 1971 and 2022. Of these, 155 (70.5%) were male with a median age of 50 years. There were 27 (12.3%) patients under 18 years of age, 6 (2.7%) individuals younger than one year, and the youngest reported case was only 20 days old. The most frequently reported symptoms on admission were fever (75.5%) and abdominal pain (66.4%), with diverticulitis (26.5%) and acute appendicitis (22%) being the two most common causes. Pylephlebitis was caused by a single pathogen in 94 (42.8%) cases and polymicrobial in 60 (27.2%) cases. However, the responsible pathogen was not identified or not reported in 30% of the included patients. The most frequently isolated bacteria were *Escherichia coli* (25%), *Bacteroides* spp. (17%), and *Streptococcus* spp. (15%). The treatment of pylephlebitis consists initially of broad-spectrum antibiotics that should be tailored upon bacterial identification and continued for at least four to six weeks after symptom presentation. There is no recommendation for prescribing anticoagulants to all patients with pylephlebitis. However, they should be administered in patients with thrombosis progression on repeat imaging or persistent fever despite proper antibiotic therapy to increase the rates of thrombus resolution or decrease the overall mortality, which is approximately 14%.

## 1. Introduction

Pylephlebitis, defined as infective suppurative thrombosis of the portal vein, is a severe condition characterized by significant morbidity and mortality, which develops as a complication of intra-abdominal suppurative processes. Pylephlebitis was first described by William Osler in 1882, who reported the case of a 28-year old patient who died of complications of septic pylethrombosis [1]. The condition, universally fatal in the pre-antibiotic era [2], has become treatable in most cases due to the advent of new diagnostic/therapeutic strategies and increased awareness of the condition. 

Due to the low incidence of pylephlebitis, no current randomized controlled trials or prospective studies describe the risk factors, diagnosis, and, most importantly, therapeutic strategies. Hence, the present systematic review aimed to investigate the etiology behind pylephlebitis in terms of pathogens involved and causative infective processes, and to report the most common symptoms at clinical presentation by analyzing data derived from case reports and series systematically as defined by Preferred Reporting Items for Systematic Reviews and Meta-Analyses (PRISMA) guidelines [3].

We performed a systematic review of the literature following the PRISMA guidelines [3] by searching the MedLine/Pubmed database for articles reporting cases of pylephlebitis using the following search terms: “septic portal vein thrombosis”, “pylephlebitis”, “suppurative portal vein thrombosis”, “portal pyemia”. All case reports and case series published in English from 1970 to 30 September 2022 were analyzed. We included all the reports in which a definitive diagnosis (PVT on imaging and positive cultures) or highly probable diagnosis (PVT on imaging and identifiable infectious focus) was present. We excluded those reports that did not include sufficient information or cases involving animals. 

**Figure 1:**
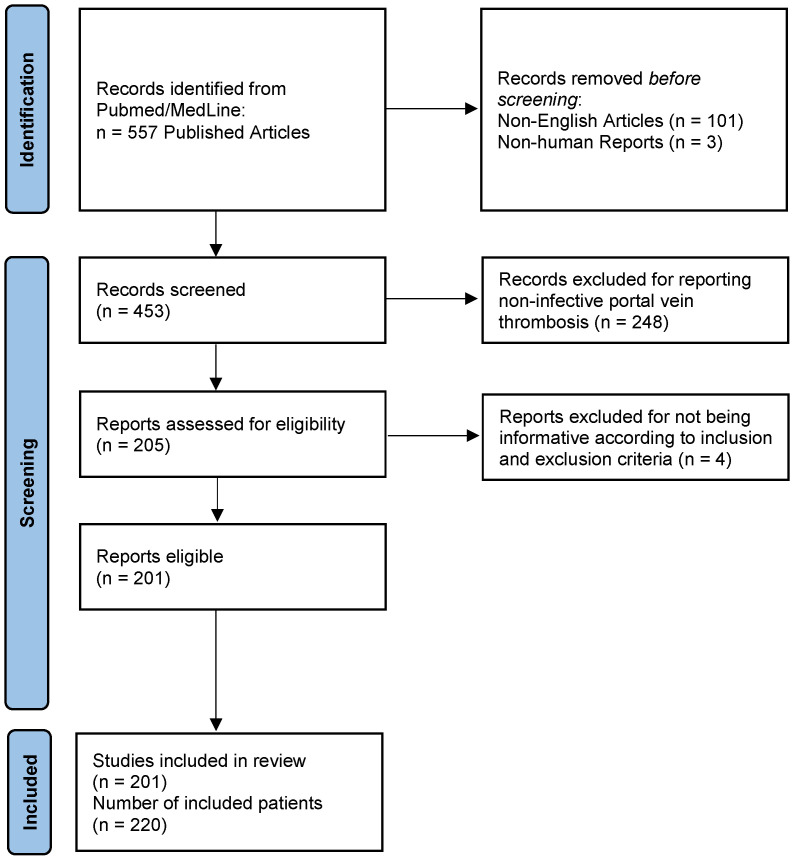
Identification of studies via databases and registers following Preferred Reporting Items for Systematic Reviews and Meta-Analyses (PRISMA) guidelines.

**Figure 2:**
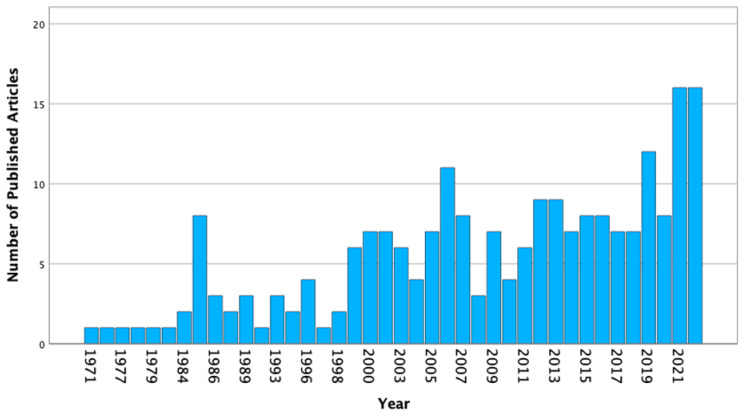
Yearly distribution of published case reports or case series involving pylephlebitis between 1971 and 2022.

The final number of articles included was 201, which resulted in a total number of 220 patients [4,5,6,7,8,9,10,11,12,13,14,15,16,17,18,19,20,21,22,23,24,25,26,27,28,29,30,31,32,33,34,35,36,37,38,39,40,41,42,43,44,45,46,47,48,49,50,51,52,53,54,55,56,57,58,59,60,61,62,63,64,65,66,67,68,69,70,71,72,73,74,75,76,77,78,79,80,81,82,83,84,85,86,87,88,89,90,91,92,93,94,95,96,97,98,99,100,101,102,103,104,105,106,107,108,109,110,111,112,113,114,115,116,117,118,119,120,121,122,123,124,125,126,127,128,129,130,131,132,133,134,135,136,137,138,139,140,141,142,143,144,145,146,147,148,149,150,151,152,153,154,155,156,157,158,159,160,161,162,163,164,165,166,167,168,169,170,171,172,173,174,175,176,177,178,179,180,181,182,183,184,185,186,187,188,189,190,191,192,193,194,195,196,197,198,199,200,201,202,203,204] as shown in Figure 1. The yearly publication rates of the included records are displayed in Figure 2. 

## 2. Epidemiology and Etiology

Pylephlebitis has a low incidence of approximately 0.37–2.7 cases per 100,000 person-years [205,206]. Interestingly, the number of reported cases has increased over time (Figure 2), with only 43 patients (19.5%) reported between 1971 and 1999, 60 cases (27.3%) between 2000 and 2009, and 117 cases (53.2%) between 2010 and 2022. Of the 220 included individuals, 155 (70.5%) were male with a median age of 50 years, as reported in Table 1. There were 27 (12.3%) patients under 18 years of age, 6 (2.7%) individuals younger than 1 year, and the youngest reported case was only 20 days old. The diagnosis of liver cirrhosis was never reported in patients published before 2010 [207] but was present in approximately 4.9% of cases published between 2010 and 2022 [208]. 

Pylephlebitis can virtually complicate any intra-abdominal or pelvic infections that develop within areas drained by the portal venous circulation. The portal vein usually originates from the union of the superior mesenteric vein with the splenic veins. It drains most of the gastrointestinal tract’s intra-abdominal portions, except the rectum’s lower part. The process behind pylephlebitis starts with localized thrombophlebitis of small veins surrounding an infected area, which then extend or migrate to the main branches of the portal vein. 

The anatomy of the portal system explains why diverticulitis (26.5%) and acute appendicitis (22%) are the two most common causes of pylephlebitis. Patients with diverticulitis had a median age of 58 years, while patients with acute appendicitis had a median age of 22.5 years, suggesting that acute appendicitis is the most prevalent cause of pylephlebitis in younger patients, while diverticulitis is the most prevalent cause in elderly patients. 

Contiguous infection can also lead to pylephlebitis, such as cholangitis/cholecystitis (3.5%) or pancreatitis (5.5%). Hepatic abscesses were found in 17 (8.5%) of cases. However, in these cases is challenging to determine whether hepatic abscesses are the cause or the consequence of pylephlebitis. It is worth highlighting that in 11.5% of cases, an apparent source of infection has not been identified or reported. Other less common causes of pylephlebitis are summarized in Table 1.

Previous studies have discussed the role of a hypercoagulable state in the onset of pylephlebitis. For example, in a case series of 44 patients, 18 (40.9%) presented a hypercoagulable state due to clotting factor deficiency, malignancy, or HIV infection [116,209]. In contrast, only 11% of patients tested for coagulation disorders were found to have a hereditary coagulation disorder [210]. 

### Microbiology

Pylephlebitis was caused by a single pathogen in 94 (42.8%) cases and polymicrobial in 60 (27.2%) cases as shown in Table 1. A pathogen was not identified or not reported in 30% of the included cases. Previous studies reported positive blood cultures in 23–62.1% of cases [205,207,208]. Even though infections caused by multiple pathogens can present worse clinical outcomes [211], a recent study failed to demonstrate worse clinical course, duration of hospitalization, therapy, and outcomes in patients with polymicrobial infection [212]. In addition, Jevtic et al. reported that polymicrobial infection did not increase mortality risk [208]. 

The most frequently isolated bacteria were *Escherichia coli* (25%), *Bacteroides* spp. (17%), and *Streptococcus* spp. (15%). Previous studies with fewer included patients show conflicting results with the three species of bacteria isolated in equal numbers [205,206,207]. *E. coli*, in particular, is a commensal bacterium of the gastrointestinal system and is often isolated in patients with acute diverticulitis, thus resulting in a not surprisingly higher prevalence in pylephlebitis. Moreover, as reported in Table 1, pylephlebitis can be caused by fungal infections (3.5%), all caused by *Candida* spp., or parasitic infections (2%), caused by intestinal worms or amoebas. 

## 3. Clinical Manifestation

The most frequently reported symptoms on admission were fever (75.5%) and abdominal pain (66.4%), as shown in Table 1. In a case series of 44 patients, all reported these two symptoms [209]. However, in a smaller series of 19 patients, 100% had a fever, while only 74% presented with abdominal pain. In another study of 67 individuals, 87% had abdominal pain, and 70% had a fever. Other common symptoms include nausea or vomiting (25.5%) and diarrhea (17.3%). Clinical signs may include right upper quadrant or generalized abdominal tenderness, hepatomegaly, and splenomegaly. Jaundice is reported in 12.7% of patients with pylephlebitis and is considered unusual in this clinical context unless there is concomitant cholangitis or liver abscesses. However, all these symptoms are common in intra-abdominal infections, which are the causative factor for the development of pylephlebitis, thus resulting in challenges to differentiate between symptoms caused by pylephlebitis or by the original infective process that caused pylephlebitis in the first place. Interestingly, in most of the reported cases, imaging leading to pylephlebitis diagnosis was performed to investigate the presence of intra-abdominal infective foci, with physicians not suspecting pylephlebitis and incidentally discovering portal vein involvement [208].

### Laboratory Findings

Leukocytosis is a common finding (80–89.7%) in pylephlebitis [207,208], even if both a normal leucocyte count [44,48,65] and neutropenia [213] were reported. Besides, most patients show high C-Reactive Protein (CRP) and erythrocyte sedimentation rate (ESR), 90.9% and 85.7%, respectively [207,208]. Elevated serum transaminases are detected in 69–71.6% of individuals [207,208], together with three- to fourfold increases in alkaline phosphatase [2,41,98,213] or five- to tenfold increases in gamma-glutamyl transferase [86,98], that can be detected in up 40% of patients. Hyperbilirubinemia, with values of total bilirubin that could reach a six-fold increase, is present in 55–74.6% of patients [207,208]. Other common laboratory findings include anemia, hypoalbuminemia, and thrombocytopenia [207,208]. 

## 4. Diagnosis

Pylephlebitis diagnosis requires the confirmation of portal vein thrombosis in patients with fever and bacteremia [44]. The diagnosis is often delayed due to the rare incidence of pylephlebitis and presentation with nonspecific symptoms. Especially in the past, the diagnosis was made by laparotomic detection of portal vein thrombosis [67,72,85] or in postmortem studies [19,79,90]. However, due to the high accessibility of modern imaging techniques, portal vein thrombosis can be promptly detected.

### 4.1. Pathogen Identification

Cultures were positive in 70% of patients with pylephebitis, as reported in Table 1. However, the high rate of undetected or not reported data accounting for 30% of cases, suggests that negative blood cultures should not automatically rule out the diagnosis of pylephlebitis, especially in the presence of suppurative intra-abdominal processes and the new detection of portal vein thrombosis. Previous data showed significantly different bacteremia rates in approximately 42–62.1% of cases [205,207,208]. Consequently, blood cultures should be promptly obtained in any febrile patient with abdominal pain before starting antimicrobial therapy. However, blood culture sensitivity usually shows a maximum sensitivity of 60%, with results being laboratory dependent and available after several days. Alternative techniques, such as polymerase chain reactions (PCR) based on the prokaryote-specific 16s ribosomal RNA gene, can be used on whole blood to increase the detection rates of microorganisms in patients with bacteremia [214]. Despite there being no evidence of this technique being used in patients with pylephlebitis, it has been shown to increase the detection rates of microorganisms in patients with bacteremia [215,216]. Furthermore, in the presence of persistently negative blood cultures, pathogens can be isolated from the primary site of infection (e.g., abscesses, fluid collection, etc.). 

### 4.2. Imaging Studies 

**Figure 3:**
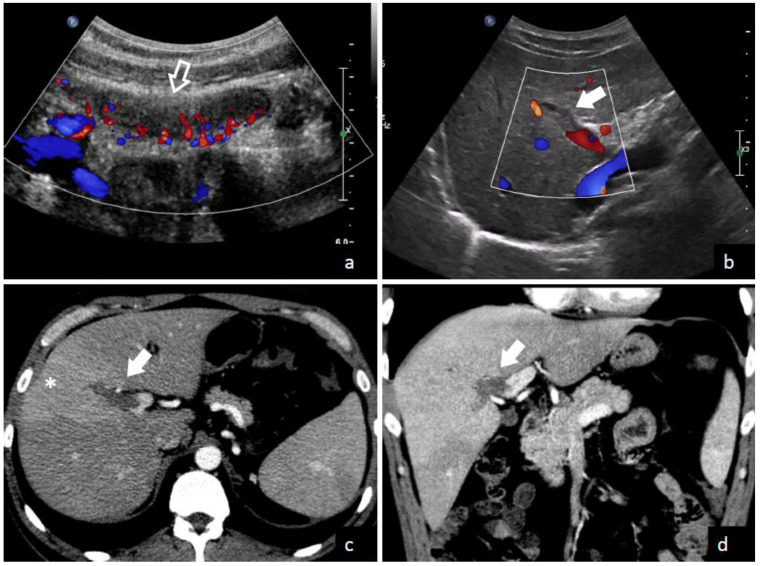
A 35-year-old male with fever and severe abdominal pain was admitted to the emergency department of Trieste University Hospital. Ultrasound with color flow Doppler images (**a**,**b**) shows acute inflammation of the appendix (empty arrow in (**a**)) and echogenic material inside an intrahepatic branch of the portal vein without color flow signal (solid arrow), due to thrombosis. Contrast-enhanced CT images in axial (**c**) and coronal (**d**) plains confirm a filling defect in a right intrahepatic portal vein branch (solid arrows), with transient hepatic attenuation differences—THAD—(* in **c**) due to hepatic arterial compensatory flow in the corresponding segment.

**Figure 4:**
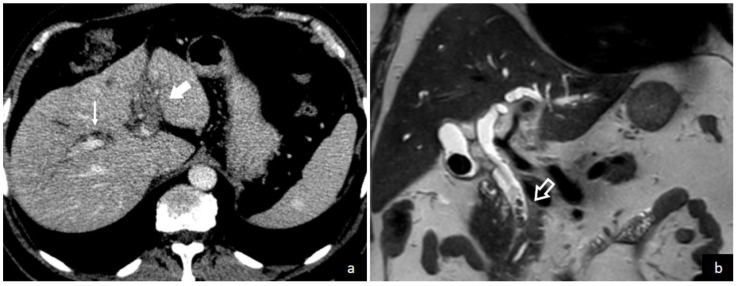
A 68-year-old male was admitted to the emergency department of Trieste University Hospital with symptoms and laboratory tests consistent with hepato-cholangitis. Contrast-enhanced CT image (**a**) shows the absence of opacification of the left intrahepatic portal vein branch (solid arrow) due to portal vein thrombosis and mild biliary dilation (thin arrow). Coronal T2 weighted MR image (**b**) demonstrates multiple filling defects (empty arrow) in the distal common bile duct consistent with choledocholithiasis and biliary sludge.

The demonstration of thrombus in the portal vein is the central finding leading to a pylephlebitis diagnosis. Both computed tomography (CT) scanning and abdominal ultrasonography can detect the presence of thrombi in the portal vein as shown in Figure 3 and Figure 4. Ultrasonography can detect the presence of echogenic material in the portal vein lumen, which can be confirmed by flow alterations on Doppler analyses [2,213]. CT scan should be preferred because of its higher definition and the additional investigative ability to identify possible abdominal or pelvic infective foci [2,39,65]. According to a recent systematic review, which enrolled studies between 2010 and 2021, the diagnosis was determined with a CT scan in 89.3% of patients and an ultrasound examination in 38.8% of patients [208]. In contrast, a previous review which included studies before 2010, found that CT scan was used only in 51% of patients. Magnetic resonance imaging (MRI) [44,210], angiography [69,91,217], endoscopic ultrasound [81], or positron emission tomography (PET) [5,42] can also be used to demonstrate portal vein thrombosis; however, their application remains limited to selected cases.

In terms of thrombus extension, localization in the main portal vein branch was detected in 57.3%, with involvement of intrahepatic branches in approximately 39% of cases (portal vein right branch in 29.1% and left branch in 24.3% of cases) [207,208], with occlusive thrombosis reported in 25.2% of patients [208]. Extension to the superior mesenteric, splenic, and inferior mesenteric veins was detected in 38–42%, 12–12.6%, and 2–9.7% of cases, respectively [207,208]. Intravascular air can be visualized in the portal system of 18% of patients [207], and its detection may precede thrombus formation. In some cases, thrombosis may not initially be evident and may result in repeated imaging from 48 h [138] to two weeks [170] after symptoms onset. 

## 5. Treatment

The treatment of pylephlebitis consists of broad-spectrum antibiotics, with the choice of empiric antibiotics depending on the most probable source of infection and the most likely involved organisms, regardless of the presence of bacteriemia. Pylephlebitis is often a polymicrobial infection, with gram-negative and gram-positive, aerobes and anaerobes bacteria, especially *E. Coli*, *Streptococcus* spp., and *Bacteroides* spp. According to previous reports, antibiotics were administered in almost all patients (94.6–100%) [205,207,208,210]. However, pylephlebitis being an uncommon infection, no randomized control studies have investigated the preferred empiric antibiotic regimens. 

Patients should be initially treated with parental antibiotics. Appropriate antibiotic regimens are reported in Table 2 [218]. These wide-spectrum regimens should be adjusted upon culture results after antimicrobial susceptibility tests are available. The duration of antibiotic therapy should at least be continued from four to six weeks after symptom presentation, continuing intravenous administration until there is a significant clinical response [48]. The remaining therapy can be administered orally with a combination of metronidazole and fluoroquinolones.

In addition to antibiotic therapy, no prospective randomized control trial establishes the utility of anticoagulants in pylephlebitis, with most of the available evidence originating from small observational studies. The rationale behind anticoagulation in pylephlebitis is to prevent thrombus extension and favor thrombus resolution. Moreover, some highly prevalent bacteria in pylephlebitis, such as *Bacteroides* spp., seem to promote coagulation by producing enzymes that degrade heparin or by promoting fibrin clotting by bacterial surface components [219]. Administration rates of anticoagulant therapy in pylephlebitis have increased in recent years, being prescribed approximately in 76.7–82% of patients [205,208], compared to previous lower rates ranging from 35% to 70% of patients [207,210]. Previous studies have found that anticoagulant administration can increase the rates of portal vein recanalization (58% vs. 21%) and reduce complications associated with chronic portal hypertension [205,207,210]. In addition, one report of 100 patients showed that anticoagulant therapy had lower mortality rates (6%) if compared to those without anticoagulant administration (22%) [207]. Similarly, Baril et al. reported that none of the patients treated with anticoagulants died compared to those who were not anticoagulated [209]. 

There is also no recommendation for prescribing anticoagulants in all patients with pylephlebitis [218]. However, they should be administered in patients with thrombosis progression on repeat imaging or persistent fever despite proper antibiotic therapy [218]. In addition, the presence of thrombosis extension beyond the portal vein main branch during the initial evaluation or confirmed hypercoagulable state are possible indications for anticoagulation [218]. No clear indications are provided in terms of the anticoagulation therapeutical approach. Usually, anticoagulation should be initiated with low-molecular-weight heparin (LMWH), and, if needed, the patient can be discharged by switching to an oral anticoagulant. The actual duration of anticoagulation therapy varies amongst studies, ranging from 128 to 143 days [205,208] to life-long treatment [116,195]. 

More invasive therapeutical approaches were performed in a relatively small portion of patients, including intraportal antibiotic administration [4,89], aspiration/drainages of pus [4,61,62,95,99], and interventional vascular procedures (such as thrombectomy or vascular bypass) [40,104,220]. 

## 6. Complications and Mortality

Thrombosis can persist, resolve, or result in a cavernous portal vein transformation characterized by veins forming within or around the thrombotic segment [117,142]. The primary complications of pylephlebites are caused by hematogenous dissemination of the pyogenic portal infection, causing metastatic abscesses. Pyogenic liver abscesses can complicate up to 37% of cases of pylephlebitis [2,102,104,207]. Other rarer sites include metastatic abscesses in the lung [145] and the brain [114].

Intestinal ischemia has also been rarely described, with one case of intestinal infarction requiring bowel resection [195,207]. Long-term complications include portal hypertension [207,213] with dilated splenic veins and the development of venous collaterals in the hepatoduodenal ligament [65]. 

As shown in Table 1, 28 patients (14%) of the 220 enrolled individuals died. Previous systematic reviews showed that mortality rates could range from 8.7% to 19% [205,207,208,210], with overall mortality < 10% for patients diagnosed after 2010 [208]. This significant decrease in mortality might suggest that the identification and treatment of pylephlebitis have improved over time. Sepsis has been found to be the cause of death in approximately 88.9% of patients [221], increasing the risk of death 17-fold [208]. Positive blood cultures were also found to be an independent risk factor for death (approximately 2.2-fold) [208]. These data confirm the fact that the primary therapy for pylephlebitis is related to antibiotic administration to prevent bacteremia and sepsis consequences.

## 7. Conclusions

**Figure 5:**
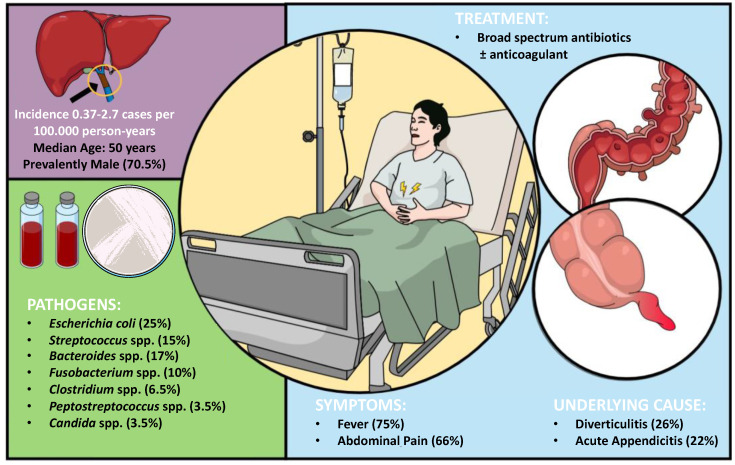
Summary of main findings on pylephlebitis in terms of incidence, pathogens, symptoms, underlying cause, and treatment.

As summarized in Figure 5, pylephlebitis is a rare condition with an incidence of 0.37–2.7 cases per 100,000 person-years, which can virtually complicate any intra-abdominal or pelvic infections that develop within areas drained by the portal venous circulation. It seems to affect mostly male individuals (70.5%) with a median age of 50. However, children and infants are not spared, considering that the youngest reported case was only 20 days old. The most frequently reported symptoms on admission were fever (75.5%) and abdominal pain (66.4%), with diverticulitis (26.5%) and acute appendicitis (22%) being the two most common causes. Pylephlebitis was caused by a single pathogen in 94 (42.8%) cases and polymicrobial in 60 (27.2%) cases. However, any pathogen was not identified or not reported in 30% of the included patients. The most frequently isolated bacteria were *Escherichia coli* (25%), *Bacteroides* spp. (17%), and *Streptococcus* spp. (15%). The imaging test of choice is abdominal contrast-enhanced CT. The treatment of pylephlebitis consists initially of broad-spectrum antibiotics that should be tailored upon bacterial identification and continued for at least four to six weeks after symptom presentation. There is no recommendation for prescribing anticoagulants to all patients with pylephlebitis. However, they should be administered in patients with thrombosis progression on repeat imaging or persistent fever despite proper antibiotic therapy to increase the rates of thrombus resolution or decrease the overall mortality, which is approximately 14%. Sepsis was the cause of death in 88.9% of cases, thus implying that prompt treatment is imperative.

## Figures and Tables

**Table 1:** Reported patients’ general characteristics, clinical presentation, mortality, detection of infective source, and pathogen isolation.

General Characteristics	
**Median Age, years** **50 (29;63)**	**Gender, *n* (%)** Male, 155 (70.5%) Female, 65 (29.5%)
**Symptoms on hospital admission, *n* (%)**	
Fever, 166 (75.5%) Abdominal Pain, 146 (66.4%) Nausea/Vomiting, 56 (25.5%) Diarrhea, 38 (17.3%)	Jaundice, 28 (12.7%) None, 10 (4.5%) Weight Loss, 3 (1.4%)
**Mortality, *n* (%)** Death, 28 (14%)	
**Data Regarding Infection Source and Pathogens**	
**Possible Source of Infection, *n* (%)**	
Diverticulitis, 53 (26.5%) Acute appendicitis, 44 (22%) None/not reported, 23 (11.5%) Liver abscess, 17 (8.5%) Gastroenteritis, 13 (6.5%) Surgery, 12 (6%) Pancreatitis, 11 (5.5%) Inflammatory bowel diseases, 8 (4%) Foreign body, 7 (3.5%) Cholangitis/cholecystitis, 7 (3.5%) Malignancy, 3 (1.5%) Gastric ulcer 3 (1.5%) Ischemia, 2 (1%)	Umbilical catheter, 2 (1%) Polypectomy, 2 (1%) Invasive liver procedures, 2 (1%) Retroperitoneal abscess, 2 (1%) Renal abscess, 1 (0.5%) Endometriosis, 1 (0.5%) Splenic abscess, 1 (0.5%) Endoscopic variceal ligation, 1 (0.5%) Cholecystocolonic fistula, 1 (0.5%) Dental abscess, 1 (0.5%) Urinary tract infections, 1 (0.5%) Burns, 1 (0.5%) Prostate biopsy, 1 (0.5%)
**Detected Microorganisms, *n* (%)**	
***Not detected/Reported, 66****(30%)* ***Aerobes*** *Gram-positive* *Streptococcus* spp., 30 (15%) *Staphylococcus* spp., 8 (4%) *Enterococcus* spp., 3 (1.5%) *Gram-negative* *Escherichia coli*, 50 (25%) *Klebsiella* spp., 9 (4.5%) *Pseudomonas aeruginosa*, 2 (1%) *Eikenella corrodens*, 2 (1%) *Acinetobacter* spp., 2 (1%) *Campylobacter jejuni*, 2 (1%) *Aeromonas hydrophila*, 2 (1%) *Morganella morganii*, 2 (1%) *Leptospira* spp., 1 (0.5%) *Proteus* spp., 1 (0.5%)	***Anaerobe****s* *Bacteroides* spp., 34 (17%) *Clostridium* spp., 13 (6.5%) *Fusobacterium* spp., 20 (10%) *Peptostreptococcus* spp., 7 (3.5%) *Lactobacillus* spp., 1 (0.5%) *Propionibacterium acnes*, 1 (0.5%) *Granulicatella adiacens*, 1 (0.5%) *Parvimonas micra*, 1 (0.5%) ***Fungi*** *Candida* spp., 7 (3.5%) ***Helminth****s* *Fasciola hepatica*, 1 (0.5%) *Strongyloides stercoralis*, 1 (0.5%) ***Amoebas*** *Amoeba* spp., 1 (0.5%) *Entoamoeba hystolytica*, 1 (0.5%)
**Number of different bacteria detected, *n*** (%) Not detected/reported, 66 (30%) One, 94 (42.8%) Two, 32 (14.5%) More than two, 28 (12.7%)	

**Table 2:** Suggested broad-spectrum antibiotic regimens in pylephlebitis.

Combination Therapy
Metronidazole (500 mg every 6–8 h), **PLUS ONE** of the following: Ceftriaxone (2 g daily) Cefotaxime (2 g every 6 h) Ciprofloxacin (400 mg every 8–12 h) Levofloxacin (750 mg daily)
**Monotherapy**
Based on beta-lactam/beta-lactamase inhibitor, with **ONE** of the following: Piperacillin-tazobactam (4.5 g every 6–8 h or 13.5–18 g in continuous infusion) Ampicillin-sulbactam (3 g every 6 h) *or* Based on carbapenem, with **ONE** of the following: Imipenem (500 mg every 6 h) Meropenem (1 g every 8 h) Ertapenem (1 g daily)

## Data Availability

Data are available online from each case report used in the analysis (check references).
